# Stability, disperse and rheological properties of oil-in-water emulsions prepared using poloxamer 188–xanthan gum mixtures

**DOI:** 10.1039/d6ra01806h

**Published:** 2026-07-02

**Authors:** Dejan Ćirin, Nebojša Pavlović, Ivana Nikolić, Jovana Milutinov, Dragana Zaklan, Milica Atanacković Krstonošić, Veljko Krstonošić

**Affiliations:** a University of Novi Sad, Faculty of Medicine, Department of Pharmacy Hajduk Veljkova 3 21000 Novi Sad Serbia nebojsa.pavlovic@mf.uns.ac.rs; b University of Novi Sad, Faculty of Technology Bulevar Cara Lazara 1 21000 Novi Sad Serbia

## Abstract

Oil-in-water (o/w) emulsions are essential systems in pharmaceutical, cosmetic, and food industries, with an emerging role in tissue engineering and regenerative medicine. In this work, mixtures of poloxamer 188 (P188), a nonionic surfactant with a unique structure and biological properties, and xanthan gum (XG), a well-known natural thickener, were employed for the development of the stable 30% (w/w) sunflower o/w emulsions, with a rotor-stator homogenizer, during the storage period set at 21 days. To evaluate the potential of P188 and XG mixtures to prepare stable emulsions, surface tension properties of their aqueous solutions, creaming stability, disperse, and rheological properties of the emulsions were studied. The investigation of the surface-tension properties indicated that there are no attractive interactions between these compounds. The analysis of the creaming stability during the storage period showed that the addition of XG in concentrations below 0.10% increased gravitational separation, most probably due to the effect of depletion flocculation. The most stable emulsions had XG in concentrations of 0.20% and higher, well above the overlap concentration of the thickener. The droplet size and size distribution analysis, performed 1 and 21 days after the emulsion preparation, showed less significant change in the Sauter mean diameter and size distribution during the storage in emulsions with XG concentrations of 0.20% and higher. The rheological investigations indicated that XG in concentrations above 0.20% yields emulsions with a semi-structured viscoelastic matrix capable of resisting flow, deformation, and creaming. All possible mechanisms involved in the observed phenomena in this work were thoroughly discussed.

## Abstract 2

Poloxamer 188–xanthan gum mixtures enable the formation of stable oil-in-water emulsions. Xanthan gum concentrations ≥ 0.20% create a semi-structured viscoelastic network that suppresses creaming and limits droplet-size changes during storage.

## Graphical abstract

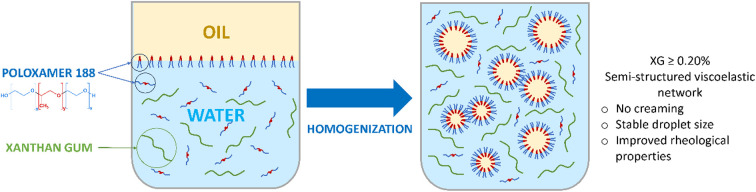

## 1 Introduction

Oil-in-water (o/w) emulsions are important systems for the pharmaceutical and cosmetic industries. They are easily applied to the skin, can be fine-tuned to provide the desired texture and spreadability, and can carry high amounts of both hydrophilic and lipophilic active compounds, thereby improving drug absorption with good patient acceptability.^1–3^ The emulsions also have an emerging role in tissue engineering and regenerative medicine.^4,5^ However, they are inherently unstable because of the immiscibility of the two phases, oil and water, and the density difference between them. To produce o/w emulsions with prolonged stability and optimize their rheological properties, mixtures of emulsifiers and stabilizers, *i.e.*, surfactants and macromolecules, are frequently investigated.^6,7^

**Fig. 1:**
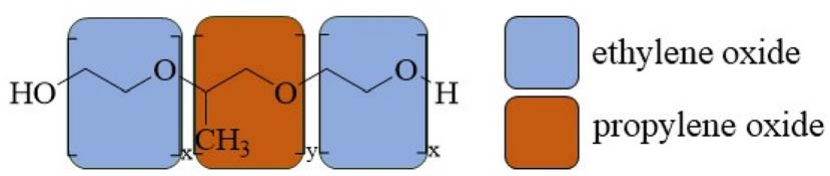
Schematic structure of poloxamer 188 (P188). Letter *x* represents the number of repeating ethylene oxide units on both hydrophilic sides of the molecule, while letter *y* represents the number of repeating propylene oxide units in the central, hydrophobic part of the molecule.

Poloxamer 188 (P188) is a hydrophilic nonionic surfactant with a distinctive structure compared to classic nonionic tensides. It is a triblock copolymer, composed of two hydrophilic polyoxyethylene (PEO) blocks, composed of ethylene oxide (EO) units, flanking a centrally positioned lipophilic polyoxypropylene (PPO) chain having propylene oxide (PO) units (Fig. 1). It has a high hydrophilic–lipophilic balance (HLB) value of 29, an average molar mass of around 8400 g mol^−1^,^8,9^ and a high value of critical micelle temperature (CMT) above 36 °C.^10,11^ P188 is a biocompatible compound, approved by the United States Food and Drug Administration (FDA) as a pharmaceutical and cosmetic excipient,^8,9^ and an ingredient in over-the-counter products.^12^ It is listed in both the US and the EU pharmacopoeia.^13^ In pharmaceutical and cosmetic products, such as micellar solutions, lotions, and creams, poloxamers are primarily used as solubilizers, emulsifiers, and cleansing agents.^14–18^ The triblock copolymer has been under the research spotlight lately due to its biological properties. Namely, P188 is a well-known membrane stabilizer. It can augment the sealing of cell membranes in various cell types, including muscle cells, fibroblasts, and endothelial cells.^19–21^ Furthermore, it promotes cell attachment and proliferation, indicating its applicability in tissue engineering, regenerative medicine, and other clinical fields.^22^

**Fig. 2:**
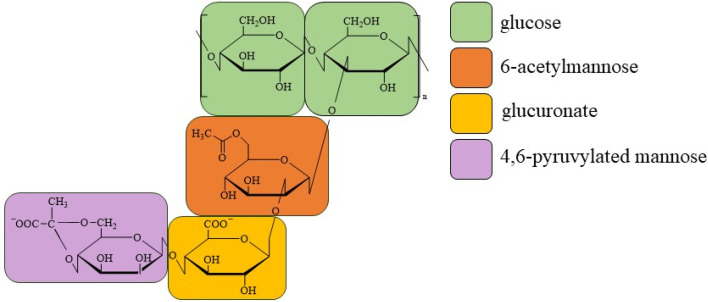
Schematic structure of xanthan gum (XG) in aqueous solution. Letter *n* represents the number of repeating units in the macromolecule.

Xanthan gum (XG) is an anionic, water-soluble, branched natural polysaccharide produced by the *Xanthomonas* bacteria genus, with a molar mass higher than 1 million g mol^−1^.^23^ XG is a polyelectrolyte in aqueous solutions, composed of a linear 1,4-linked β-d-glucose backbone, with a negatively charged trisaccharide side chain attached to every other glucose unit of the backbone,^24^ as shown in Fig. 2. It is a biocompatible compound approved by the FDA as both a food additive and a pharmaceutical excipient,^25,26^ and is frequently used in cosmetic products.^27^ In pharmaceutical and cosmetic formulations, such as lotions, creams, and toothpaste, XG is mainly used as a thickener and stabilizer.^23,28^ The polysaccharide belongs to a group of non-adsorbing macromolecules with high thickening, *i.e.*, viscosity-enhancing, properties.^25,29^ Due to this, it is used as a stabilizer of various dispersed systems, frequently in mixtures with surface-active compounds.^7,24,28^ XG can also be used to fine-tune the rheological properties of o/w emulsions. Depending on the concentration, its aqueous solutions show pseudoplastic or even thixotropic behavior.^30,31^ Its applicability in tissue engineering and regenerative medicine is also investigated.^32,33^

Sunflower oil was chosen as a model oil since it is commonly used in topical formulations as a non-comedogenic emollient in the pharmaceutical and cosmetic industries.^34,35^ It contains various bioactive compounds, such as tocopherols, sterols, squalene, and carotenoids, which are beneficial to the skin.^36,37^ As a biocompatible oil, sunflower oil has shown applicability in tissue engineering.^38^

Despite the favorable safety profile of P188 and XG, their widespread usage and emerging roles in various industries, the possible interactions between P188 and XG, as well as the applicability of these mixtures in producing stable emulsions, disperse, and rheological properties of such systems, were not investigated, according to the authors' knowledge. Therefore, the aim of this study was to gain insight into the possible interactions between P188 and XG in aqueous solutions and assess their ability to form o/w emulsions by investigating creaming stability, disperse characteristics, and rheological properties. It is envisioned that the results of this study could provide a better understanding of potential usage of hydrophilic triblock copolymers and XG mixtures in the development of emulsions and to gain more knowledge regarding mechanisms involved in their formation and stabilization, with a broader goal to speed up the development of novel pharmaceutical and cosmetic formulations as well as new matrices for tissue engineering and regenerative medicine applications.

The potential application of the present study and the investigated properties of P188/XG solutions and emulsions with sunflower oil are summarized in Table 1.

**Table 1:** Required and desirable properties of an emulsifier and a stabilizer system for pharmaceutical, cosmetic, and tissue engineering applications, together with known attributes of poloxamer 188 (P188) and xanthan gum (XG), and the investigated properties of their solutions and the emulsions

Applicability of investigated emulsions	Required and desirable attributes of an emulsifier and a stabilizer system	Investigated properties of P188 and XG solutions, and the emulsions
Pharmaceutical/cosmetic lotions and creams	• Non-toxic	• Surface tension measurements
	• Non-irritating	• Oil–water interface measurements
	• Non-sensitizing	• Flow behavior
	• Surface active	• Viscoelastic behavior
	• Adsorbs at the oil–water interface	• Change in droplet-size distribution
	• Prevents flocculation and coalescence	• Physical stability
	• Provides creaming stability	
	• Prevents phase separation	
	• Provides spreadability to emulsions	
	• Forms a viscoelastic matrix	
	• Shows shear-thinning flow behavior	
Tissue engineering/regenerative medicine	• Non-toxic	• Flow behavior
	• Biocompatible	• Viscoelastic behavior
	• Forms a viscoelastic matrix	• Change in droplet-size distribution
	• Shows shear-thinning flow behavior	• Physical stability
	• Provides creaming stability	
	• Prevents phase separation	

## 2 Materials and methods

### 2.1 Materials

Poloxamer 188 (Kolliphor® P 188, LOT number: WPDH566B) with an average molar mass of 8369 g mol^−1^, according to the certificate of analysis supplied by the producer, was donated by BASF Chemtrade GmbH (Ludwigshafen, Germany). Xanthan gum (Xantural® 180 CP), food grade, with a viscosity of 1561 mPa s for a 1% solution in 1% KCl solution, based on the certificate of analysis provided by the manufacturer, was donated by CP Kelco (Atlanta, GA, USA). Sodium azide was purchased from Sigma-Aldrich (Taufkirchen, Germany, product number: 71290) and was used as an antimicrobial preservative in the aqueous solutions and the emulsions. Sunflower oil produced by Dijamant (Zrenjanin, Serbia), was obtained in the local market. Distilled water was used for the preparation of aqueous solutions and emulsions in the study. All reagents were used without further purification.

### 2.2 Surface and interfacial tension measurements

Surface and interfacial tension measurements were performed on three sets of aqueous solutions containing pure P188 or P188/XG mixtures, at 25 ± 0.1 °C. For interfacial tension measurements, the aqueous solutions were overlaid with sunflower oil. The du Noüy ring method was used on a Krüss K20 Easy Dyne tensiometer (A.KRÜSS Optronic GmbH, Hamburg, Germany). The first set of water solutions consisted of pure P188 water solutions with the following concentration range of the nonionic surfactant: 0.0000125–5% (w/w). The second and third sets of aqueous solutions had the same concentrations of P188 as the first set, as well as XG at the fixed concentrations of 0.025 and 0.050% (w/w), respectively. All investigated water solutions contained sodium azide at a concentration of 0.02% (w/w), corresponding to 3.08 mM. The measurements for each solution were carried out four times. The obtained values were used to determine the mean surface tension or mean interfacial tension values for each solution and the standard deviation.

### 2.3 Emulsion preparation

Two sets of emulsions were prepared in this study, which differed only in the concentration of P188. The first set contained P188 in a concentration of 1% (w/w) and the second set contained the emulsifier in a concentration of 3% (w/w), calculated on the mass of the entire emulsion. The concentration of XG was in the following concentrations, in both sets: 0, 0.025, 0.050, 0.075, 0.10, 0.15, 0.20, 0.30 and 0.40% (w/w), calculated on the mass of the aqueous, continuous, phase. All the prepared emulsions contained oil phase in a concentration of 30% (w/w). The emulsions were produced using a rotor-stator homogenizer (T25 digital ULTRA-TURRAX, IKA®-Werke GmbH & Co., Staufen, Germany). The homogenization was performed at 15 000 rpm, for 10 minutes, at a temperature-controlled water bath, at a constant temperature of 25 °C. The emulsions contained 0.02% (w/w) of sodium azide, calculated on the mass of the water phase.

### 2.4 Creaming stability

Both sets of emulsions, with 1 or 3% (w/w) of P188 and following XG concentrations: 0, 0.025, 0.050, 0.075, 0.10, 0.15, 0.20, 0.30 and 0.40%, were investigated for their creaming stability. The emulsions with 0, 0.025, 0.050, 0.075, and 0.10% (w/w) of XG were transferred to graduated cylinders 5 hours after preparation, to avoid foam transfer, due to significant foaming during preparation. The emulsions with 0.15, 0.20, 0.30, and 0.40% (w/w) of XG were left for 1 day (24 hours) after preparation before transferring to graduated cylinders, due to the more stable foam formation during the preparation. All emulsions were visually monitored for 21 days after preparation. The creaming stability was evaluated by calculating the creaming index value (*H* (%)) on each day. The *H* value was calculated as the ratio of the height of a serum layer at the bottom (*H*_S_) to the total height of an emulsion sample (*H*_T_) in the graduated cylinders, according to the following formula:1
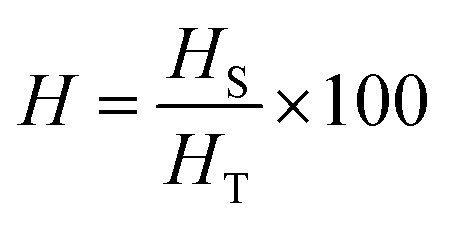


### 2.5 Emulsion droplet size and size distribution analysis

A laser diffraction particle size analyzer (Mastersizer 2000, Malvern Instruments Ltd, Worcestershire, UK) was employed to measure particle size distribution in emulsions with 3% (w/w) of P188 and 0, 0.025, 0.050, 0.075, 0.10, 0.15, 0.20, 0.30 and 0.40% (w/w) of XG. All investigated emulsions were left for 24 hours after preparation to avoid the presence of air bubbles in the emulsion samples during measurements. The measurements were carried out 24 hours and 21 days after the preparation, at least three times. The obtained results were used to calculate the mean values and standard deviations, as well as to find the size distribution. The results were analyzed using Malvern software. The refractive indices used for particle size determination were 1.47 for the oil phase and 1.33 for the aqueous phase (relative refractive index 1.10). The particle size of the investigated emulsions was presented using the Sauter mean diameter (*d*_3,2_ (µm)).

### 2.6 Rheological characterization of emulsions

Rheological testing was performed for emulsions containing 3% (w/w) of P188 and XG in the following concentrations: 0, 0.025, 0.050, 0.075, 0.10, 0.15, 0.20, 0.30 and 0.40% (w/w), calculated on the mass of the aqueous phase. The measurements were carried out using a HAAKE MARS rheometer (Thermo Scientific, Karlsruhe, Germany) equipped with a CC25 DIN/Ti cylindrical measuring system, at 25 ± 0.1 °C.

First, the flow behavior of each emulsion was evaluated by continuous hysteresis loop tests. The samples were subjected to a gradual increase of shear rate from 0.01 to 150 s^−1^ over 120 s, followed by maintaining the maximum shear rate for 60 s, and subsequently decreasing it to 0 s^−1^ during the next 120 s. The obtained flow curves were fitted to the Ostwald-de Waele power law model according to the following equation:

2 *τ* = *K

<svg xmlns="http://www.w3.org/2000/svg" version="1.0" width="10.615385pt" height="16.000000pt" viewBox="0 0 10.615385 16.000000" preserveAspectRatio="xMidYMid meet"><metadata>
Created by potrace 1.16, written by Peter Selinger 2001-2019
</metadata><g transform="translate(1.000000,15.000000) scale(0.013462,-0.013462)" fill="currentColor" stroke="none"><path d="M320 960 l0 -80 80 0 80 0 0 80 0 80 -80 0 -80 0 0 -80z M160 760 l0 -40 -40 0 -40 0 0 -40 0 -40 40 0 40 0 0 40 0 40 40 0 40 0 0 -280 0 -280 -40 0 -40 0 0 -80 0 -80 40 0 40 0 0 80 0 80 40 0 40 0 0 80 0 80 40 0 40 0 0 40 0 40 40 0 40 0 0 80 0 80 40 0 40 0 0 120 0 120 -40 0 -40 0 0 -120 0 -120 -40 0 -40 0 0 -80 0 -80 -40 0 -40 0 0 200 0 200 -80 0 -80 0 0 -40z"/></g></svg>


*^*n*^

where *τ* (Pa) represents shear stress, ** (s^−1^) is shear rate, *n* (no dimensional) is the flow behavior index, and *K* (Pa s^*n*^) is the consistency index.

To characterize the viscoelastic properties, all emulsions were subjected to amplitude sweep tests in order to determine the limits of the linear viscoelastic region (LVR). The storage modulus (*G*′) and loss modulus (*G*″) were recorded as functions of oscillatory shear stress (0.01–10 Pa) at a constant frequency of 1 Hz. Yield stress (*τ*_0_) was determined as the shear stress corresponding to a 10% deviation from the *G*′ plateau value, indicating the end of the LVR.^39^ Based on the amplitude sweep results, a representative strain within the LVR was selected for subsequent frequency sweep tests, which were performed for the emulsions exhibiting a defined LVR. In frequency sweep experiments, *G*′ and *G*″ moduli were recorded as functions of oscillation frequency (0.1–10 Hz) at a constant shear stress, previously selected according to the amplitude sweep test results.

### 2.7 Statistical analysis

The *t*-test was carried out at a significance level of 0.05 (*p* = 0.05) to determine whether a statistically significant difference existed between the obtained data. For this purpose, the one-way ANOVA procedure and Tukey's multiple comparisons test were employed, using OriginPro 2022 software (v. 9.9.0.225).

## 3 Results and discussion

### 3.1 Surface and interfacial tension properties of P188 and P188/XG mixtures

**Fig. 3:**
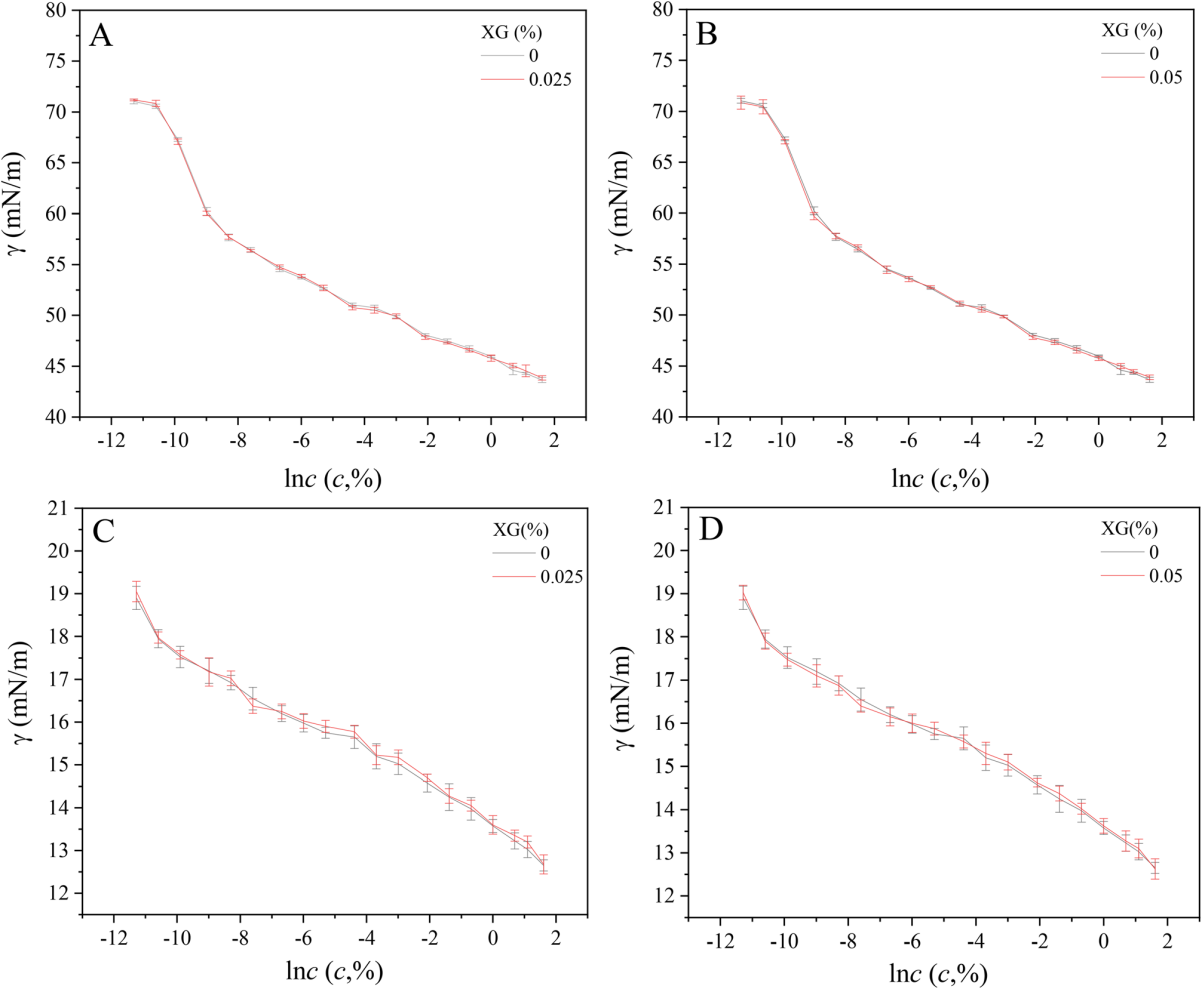
Dependence of surface tension (A and B) and interfacial tension at the oil/water interface (C and D) on the natural logarithm of poloxamer 188 (P188) concentration (w/w) for water solutions composed of P188 or P188/xanthan gum (XG) with constant XG concentration. The XG concentration was set at 0.025% (w/w) (A and C) or 0.05% (w/w) (B and D).

It is important to identify potential interactions between an emulsifier and a stabilizer to better understand the mechanisms underlying various phenomena observed during stability studies, dispersion analysis, and rheological investigations of emulsions. For this purpose, surface and interfacial tension measurements are commonly used. The presence of interactions is determined by comparing the surface or interfacial tension values obtained for aqueous solutions of a surfactant before and after the addition of a macromolecule.^6,40–42^ The results of surface and interfacial tension measurements of aqueous solutions containing P188 or P188/XG mixtures, with a constant concentration of XG, are shown in Fig. 3.

Based on the obtained results, it can be observed that with the increase in P188 concentration surface tension value of the aqueous solutions and the interfacial tension decrease. There were no statistically significant differences between the surface tension values of the water solutions in the presence or absence of XG. Also, the presence of XG did not result in statistically significant changes in the interfacial tension of the investigated systems. This indicates that there are most probably no attractive interactions between the nonionic surfactant and the polysaccharide in aqueous solution. A similar phenomenon was noticed in Triton X-100/polystyrene sulfonate mixtures, where the presence of a constant concentration of the polyelectrolyte, *i.e.*, polystyrene sulfonate, did not influence the surface activity of Triton X-100.^40^ However, the constant concentration of XG had a statistically significant influence on surface tension values of aqueous solutions of polysorbate 80 in a previous study.^41^ The higher surface tension values of polysorbate 80 water solutions in the presence of XG were attributed to weak attractive interactions between the surfactant and the polysaccharide in the bulk of the solution. Furthermore, the increase in surface tension values of a nonionic surfactant dodecyl hexaethylene glycol ether, *i.e.*, C12E6, after the addition of polystyrene sulfonate was attributed to attractive hydrophobic interactions between the surfactant and the polyelectrolyte in the bulk, leading to depletion of the surfactant at the interface.^43^ It could be hypothesized that the absence of attractive interactions between a nonionic surfactant and a polyelectrolyte, *i.e.*, P188 and XG, in our study, could be attributed to a weaker tendency of P188 molecules to form hydrophobic interactions in water solutions under the investigated conditions. Namely, in aqueous solutions of poloxamers, below their CMT, the polymeric molecules exist in the form of unimers.^10,11,44,45^ Furthermore, methyl groups of PO units of the hydrophobic part of poloxamers are hydrated at temperatures below CMT.^45^ It is also considered that ether oxygen from PO groups can form hydrogen bonds with water molecules.^10,45^ Therefore, taking into account the high CMT of P188,^10,11^ it can be hypothesized that the hydrated PPO blocks of P188 are less inclined to undergo hydrophobic interactions with XG in the investigated systems.

### 3.2 Creaming stability

Instability of emulsions arises from their thermodynamic instability and the density difference between the oil and water phases. In creaming, less dense, buoyant oil droplets of the oil-in-water emulsions rise to the top of the dispersion.^46^ This causes the formation of two distinct layers, a visible cream layer rich in oil at the top and a more or less clear serum layer at the bottom.^47^ Besides the density difference between the dispersed and continuous phases, Stokes' law shows that creaming speed depends on the particle radius and the viscosity of the continuous phase.^48^ Therefore, emulsions with smaller droplets and higher viscosity of the continuous phase tend to have lower creaming speeds. To evaluate the creaming stability of emulsions, the creaming index is typically monitored over a certain storage period.

**Fig. 4:**
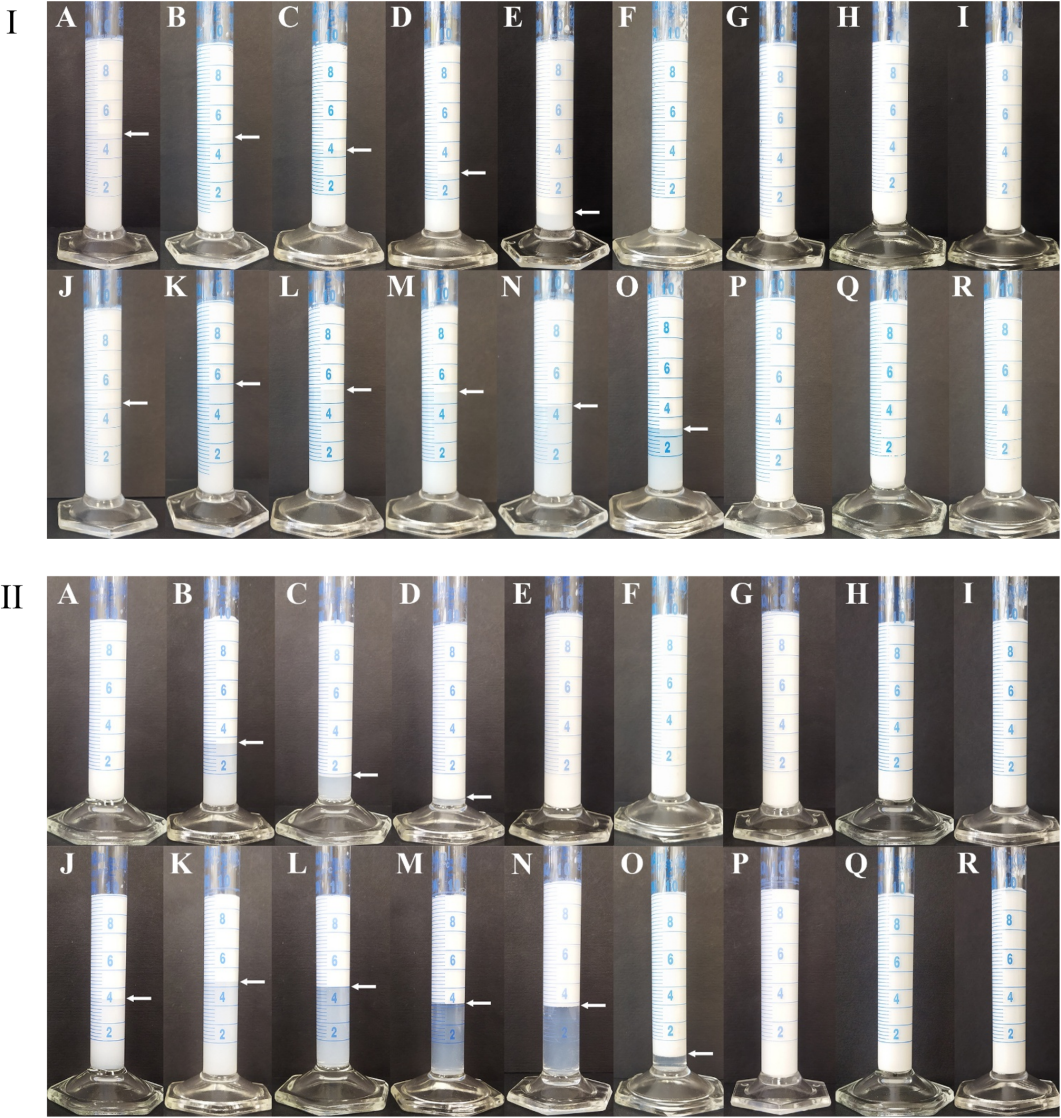
Photographs of 30% oil-in-water emulsions containing (I) 1% or (II) 3% of poloxamer 188 (P188) and the following xanthan gum (XG) concentrations: (A and J) 0%, (B and K) 0.025%, (C and L) 0.05%, (D and M) 0.075%, (E and N) 0.10%, (F and O) 0.15%, (G and P) 0.20%, (H and Q) 0.30%, (I and R) 0.40%. (A–I) were photographed after 1 day (24 hours) of storage, and (J–R) were photographed after 21 days, of storage.

In this study, the change of creaming index was observed during the storage period of 21 days, by direct observation of the dispersed systems in graduated cylinders. Photographs of the investigated o/w emulsions in graduated cylinders after 1 and 21 days of their preparation are shown in Fig. 4. By the end of the storage period, two layers, turbid at the top and rich in oil droplets, and less turbid at the bottom of the cylinder, were observed in emulsions having only P188 or P188/XG mixtures with the polysaccharide concentration below 0.2%.

**Fig. 5:**
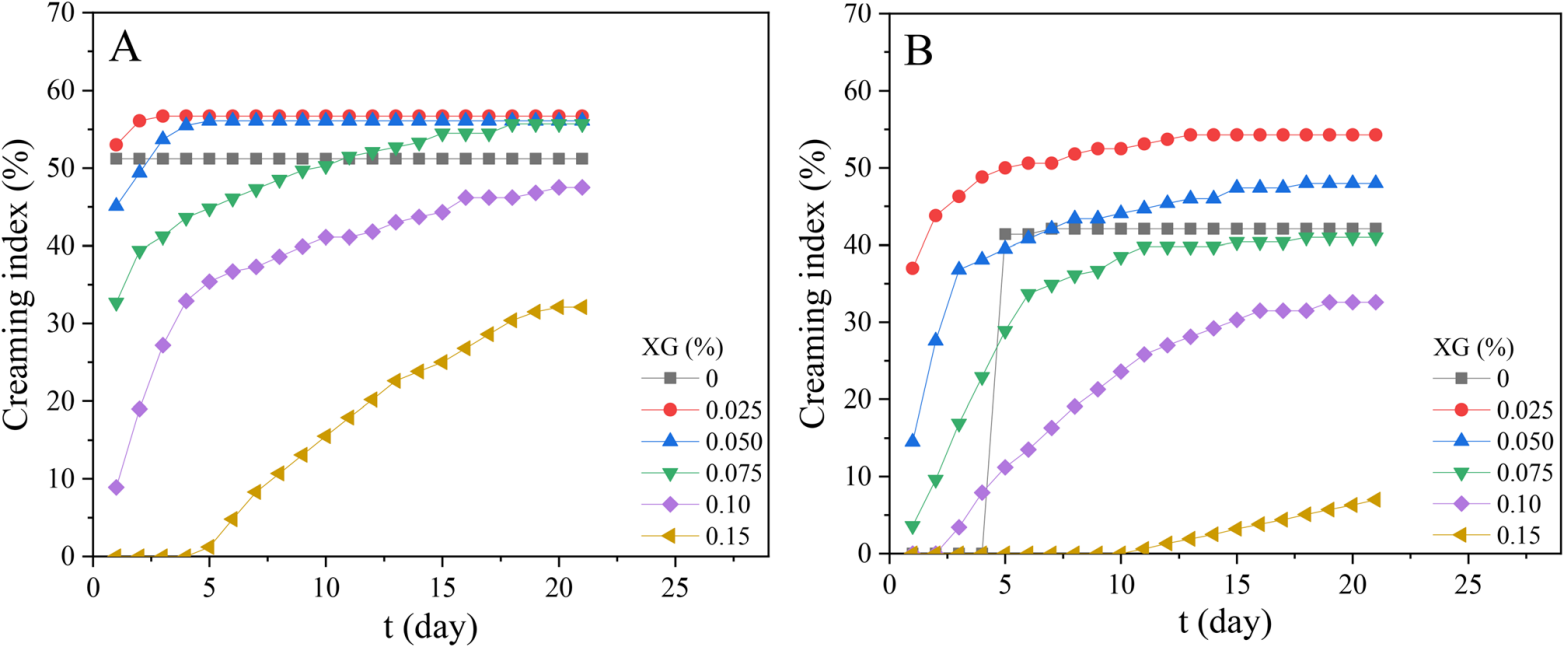
Change of the creaming index value during storage of oil-in-water emulsions containing poloxamer 188 (P188) in concentration of (A) 1% or (B) 3%, and various concentrations of xanthan gum (XG). The change in the creaming index was shown only for emulsions that exhibited gravitational separation.

To observe the creaming stability more closely, the dependence of the creaming index values on XG concentration of emulsions containing 1% or 3% of P188, during the time period of 21 days, is shown in Fig. 5. Based on the results shown in Fig. 4 and 5, it is clear that both P188 and XG concentrations influence the creaming stability of the emulsions. Emulsions with 1% of P188 were less stable than those with 3% of the nonionic surfactant, for the same concentration of XG. This is most probably because surfactants, *i.e.*, emulsifiers, are less effective at covering the surface of droplets at lower concentrations, allowing coalescence during homogenization to occur more intensively.^49^ The data also indicate that the addition of XG in concentrations below 0.10% reduces creaming stability during the storage period of 21 days. The rapid destabilization, *i.e.*, early onset of gravitational separation, was particularly observable in emulsions with 3% of P188 and 0.025–0.075% of the polysaccharide, reflected in a higher creaming index, compared to the emulsion without XG after the first 24 hours (Fig. 4 and 5). This rapid destabilization likely stems from a depletion flocculation mechanism, which is common in o/w emulsions containing a non-adsorbing macromolecule such as XG.^42,50^ Creaming was observed even in some emulsions with XG in concentrations above its overlap concentration, which is 0.08%,^51^ although at a lower rate, similarly to previous studies.^42,50^ At these concentrations, of 0.10% and 0.15% of XG, the aqueous phase was unable to prevent the gravitational separation, most probably due to insufficient viscosity, even though the overlap concentration was exceeded. However, at higher concentrations, above 0.15%, a significant improvement in emulsion stability was noticed. Emulsions with 0.20%, 0.30%, and 0.40% of XG did not show signs of creaming instability over the 21 day observation period, regardless of P188 concentration. It can be assumed that at a concentration of 0.20%, XG reached or surpassed the critical viscosity concentration in the prepared emulsions, probably as a result of stronger interactions between XG chains. Namely, once the critical viscosity concentration is reached, the onset of creaming is usually effectively delayed, despite the effect of the depletion flocculation.^42,52^

### 3.3 Droplet size and droplet size distribution of emulsions

Droplet size and size distribution are important properties of emulsions that influence their appearance, stability, rheology, and texture.^53^ In pharmaceutical emulsions, droplet size and size distribution can determine the rate of drug absorption.^47^ Generally, the emulsions with a smaller droplet size are more desirable, especially due to the greater stability.^54^ Droplet size and size distribution also indicate the effectiveness of homogenization, as this process disrupts the droplets, reducing their size. The change of droplet size and size distribution over time can also indicate the existence of instabilities in the system, such as flocculation and coalescence.^47^ Emulsifiers and stabilizers are added to emulsions to prevent these instabilities, *i.e.*, to ensure their kinetic stability. In the investigated emulsions, the task of P188 was to adsorb at the surface of the newly formed droplets and reduce interfacial tension. The adsorbed P188 should also protect against flocculation and coalescence by a steric mechanism, due to its long hydrophilic polyoxyethylene chains.^55^ XG had a role in increasing the viscosity of the continuous phase. This should prevent re-coalescence during homogenization, reduce mobility and collision frequency of the particles during the storage period, to ensure kinetic stability for an extended period.

**Fig. 6:**
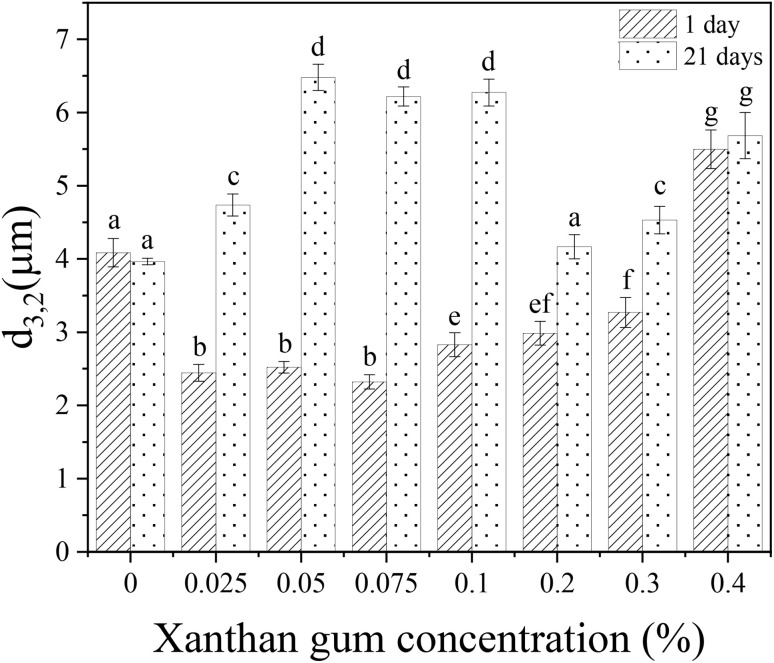
Change of the Sauter mean diameter (*d*_3,2_) of oil-in-water emulsions with 30% of oil, 3% of poloxamer 188, and various concentrations of xanthan gum, obtained after 1 and 21 days. Different letters indicate a statistically significant difference (*p* < 0.05).

**Fig. 7:**
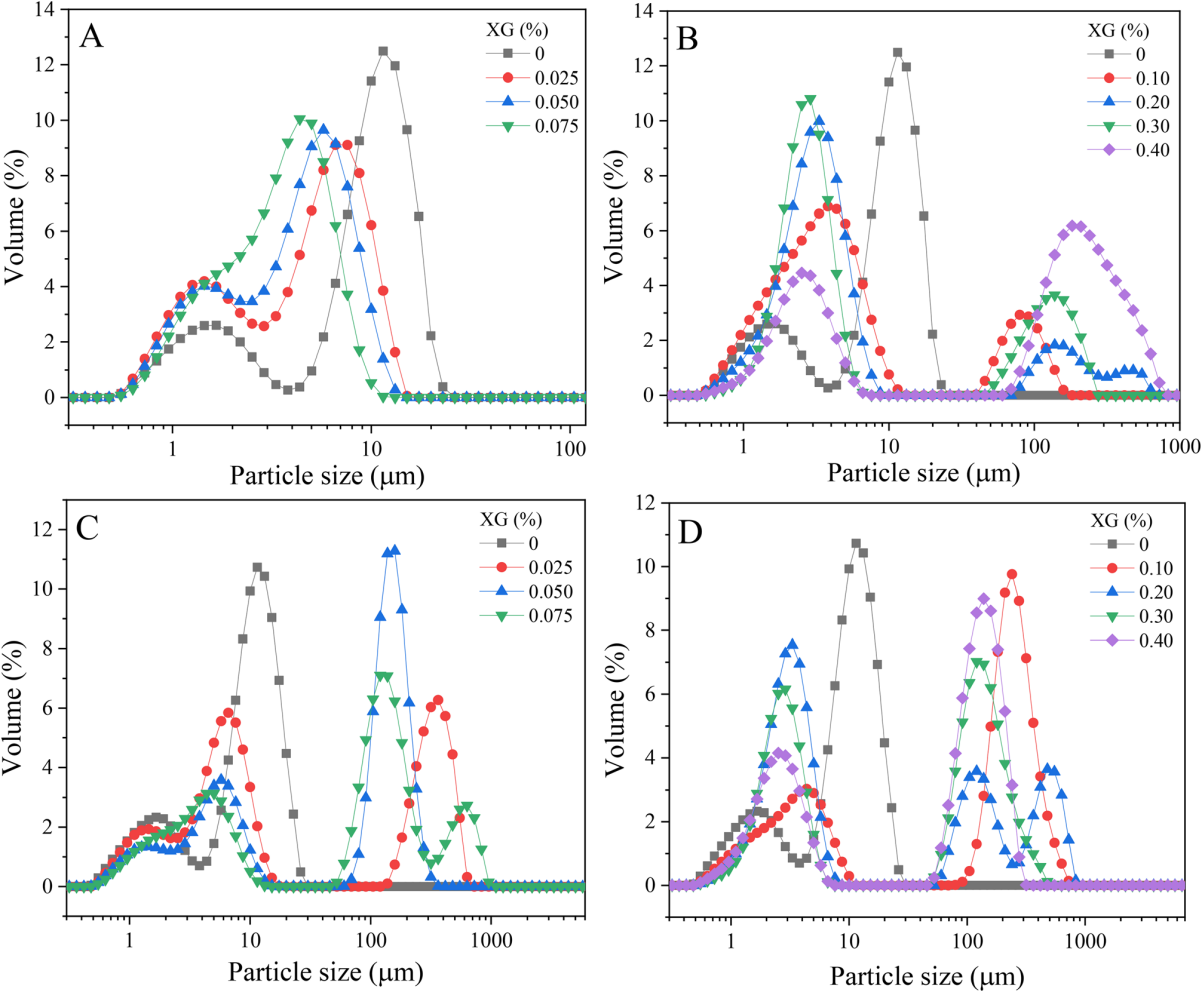
The particle size distribution of oil-in-water emulsions with 30% of oil, 3% of poloxamer 188, and various xanthan gum (XG) concentrations: (A and C) 0–0.075%, (B and D) 0.10–0.20%. (A and B) and (C and D) were obtained after 1 and 21 days, respectively.

The results obtained by investigating droplet size and droplet size distribution of prepared emulsions with 3% of P188 with or without XG are presented in Fig. 6 and 7. The droplet size was characterized using the Sauter mean diameter (*d*_3,2_).

Based on the data presented in Fig. 6, it can be noticed that with the increase in XG concentration, the mean *d*_3,2_ values of emulsions stored for 1 day decreased up to the concentration of 0.075% of XG. The decrease of the *d*_3,2_ values was characterized by a shift of the distribution curve towards the left, *i.e.*, towards the smaller values (Fig. 7(A)). The distributions were mainly bimodal, with a narrower range between the droplet sizes compared to the emulsion without XG. With further increase in XG concentration, above 0.075% of XG, the mean *d*_3,2_ values of emulsions increased (Fig. 6), but mainly remained lower compared to the emulsion prepared without the polysaccharide. The range between the droplet sizes, however, significantly increased (Fig. 7(B)).

The observed decrease in the mean droplet sizes with the initial increase in XG concentration is most probably the consequence of the increasing viscosity of the continuous phase. The increase in viscosity averts re-coalescence during homogenization, leading to smaller droplets. However, at the concentrations of XG above 0.075%, the viscosity increase probably led to less effective disruption, *i.e.*, breakdown, of the droplets during homogenization. Namely, as it was previously noticed, the XG overlap concentration is around 0.08%.^51^ Above the overlap concentration, XG polyelectrolytes begin to interact and overlap, resulting in a more significant rise in the viscosity of the continuous phase, with an increase in XG concentration.^50^ This most probably reduced homogenization effectiveness and led to the increase in mean *d*_3,2_ values for the emulsions with XG concentration above the overlap concentration and the change in the behavior of the distribution curve.

By analyzing the difference between the mean droplet size obtained after 1 and 21 days of storage (Fig. 3), it can be observed that the mean *d*_3,2_ values increased most significantly for emulsions with XG concentration below 0.20%, during storage, probably due to the extended effect of depletion flocculation. Namely, it can be hypothesized that in the first 24 hours of the storage period, depletion flocculation caused the formation of loosely packed aggregates, which were not detected as individual particles during the droplet size determination. However, after 21 days, the prolonged effect of depletion flocculation might have caused the aggregates to become denser and be recognized as individual particles. As a result, the emulsions with XG concentration of 0.025–0.10% exhibited mainly multimodal distribution after the storage period, with a wide range of droplet sizes (Fig. 7(C)). The change in the mean *d*_3,2_ values was less significant in emulsions with XG concentration of 0.20% and higher, most probably due to the high viscosity of the continuous phase. Namely, as previously observed, at XG concentration of 0.20% the critical viscosity concentration is reached or surpassed, which more significantly slows down the Brownian motion of droplets. This probably more effectively reduced the depletion flocculation in emulsions with XG of 0.20% and above, during the whole storage period. Among the investigated emulsions stabilized with P188/XG mixtures, statistically significant changes in the mean *d*_3,2_ values after 21 days of storage were observed for all investigated emulsions, apart from the emulsion containing 0.40% of XG.

### 3.4 Rheological behavior of P188–XG emulsions

All investigated emulsions with XG exhibited non-Newtonian, shear-thinning (*i.e.*, pseudoplastic) behavior, with the extent of pseudoplasticity strongly dependent on XG concentration. In the absence of XG, emulsions stabilized only with 3% (w/w) P188 showed Newtonian flow, with flow behavior indices (*n*) close to 1 and very low consistency indices (*K*). Although oil-in-water emulsions with a dispersed-phase volume fraction of 30% are generally expected to exhibit non-Newtonian behavior due to droplet–droplet interactions and structural organization within the continuous phase,^56,57^ the emulsion without XG in this study behaved as Newtonian fluids. This can be explained by the rheological profile and functional role of P188 at the concentration used. Dilute aqueous P188 solutions at 1–3% do not form micelles at room temperature^57^ and behave as low-viscosity liquids rather than structured systems. P188 is thermoresponsive, but its gelation temperature at pharmaceutically relevant concentrations is relatively high; it was reported that 20% (w/v) aqueous P188 solutions gel at temperatures above 50 °C.^59^ The P188 concentrations used in this study are therefore far below those required for thermally induced structuring, which indicates that P188 primarily functioned as an emulsifier in the emulsions, providing steric stabilization of droplets rather than contributing to the continuous phase network formation.^58^

The efficient steric stabilization may have minimized flocculation and reduced droplet–droplet interactions, suppressing the structural constraints that produce shear-thinning at higher oil fractions. This is consistent with several earlier studies showing that highly stabilized or monodisperse emulsions may deviate from the expected non-Newtonian behavior even at similar dispersed-phase fractions.^60,61^ In addition, the relatively low viscosity ratio between sunflower oil and the P188-containing aqueous phase may contribute to more Newtonian-like flow behavior at low shear rates. When the viscosity difference between the oil droplets and the continuous aqueous phase is relatively small, droplets can deform and exhibit internal circulation under shear, resulting in lower hydrodynamic resistance compared to rigid-particle systems. The relatively low viscosity of the continuous phase in P188-stabilized emulsions may also reduce droplet–droplet interactions and structural organization, thereby limiting non-Newtonian shear-thinning behavior.^62–64^

**Fig. 8:**
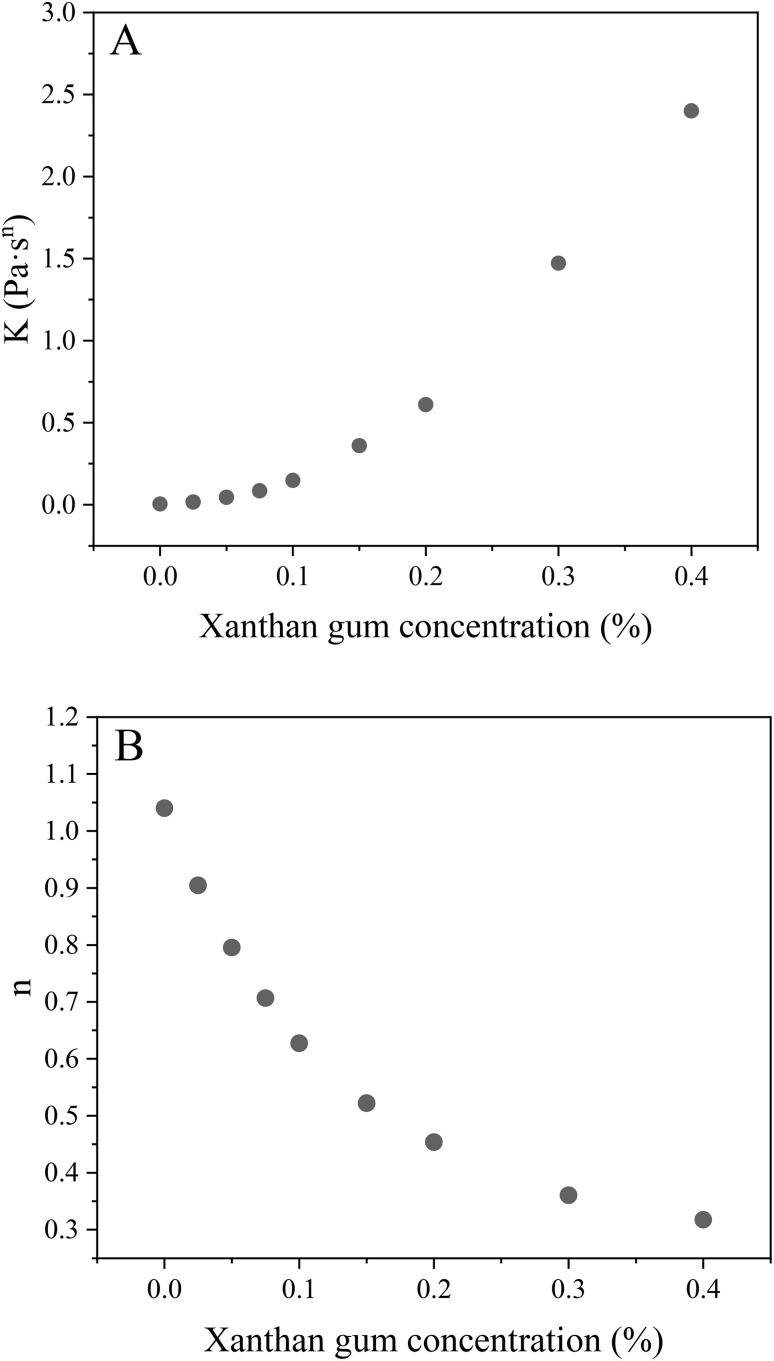
Effect of xanthan gum (XG) concentration on the steady-shear rheological parameters of emulsion containing 3% P188: (A) Consistency index (*K*) and (B) flow behavior index (*n*), obtained from fitting the flow curves to the Ostwald-de Waele model.

The addition of XG significantly changed the flow behavior (Table 2). The consistency index (*K*) increased steadily with the increase of XG concentrations (Fig. 8(A)), confirming the progressive thickening of the continuous phase. On the other hand, the flow behavior index (*n*) decreased from values close to 1 in XG-free emulsions to approximately 0.3 at the highest XG content of 0.40% (Fig. 8(B)), indicating increasingly pronounced shear-thinning behavior. These results suggest the formation of a more entangled and structured aqueous phase with an increase in XG, which is in agreement with previous observations for polysaccharide-thickened emulsions and for protein/XG systems.^7,42^ Higher XG concentrations enhance the apparent viscosity by establishing a semi-rigid, high molecular weight network, while the decreasing *n* values demonstrate stronger sensitivity to shear as the internal structure becomes more easily oriented and partially disrupted. It is well established that XG can form a semi-rigid, entangled network at higher concentrations, transitioning from a dilute polymer solution to a structured, viscoelastic matrix that significantly increases viscosity and promotes shear-thinning behavior.^65^ This ability arises from its specific molecular structure, particularly the double-helix conformation, which facilitates intermolecular associations and network formation, contributing to the gel-like characteristics of continuous phase of the emulsions with incorporated dispersed oil droplets at higher XG concentrations.^66^

**Table 2:** Consistency index (*K*) and flow behavior index (*n*) values of the emulsions obtained from Ostwald-de Waele power law model fitting and emulsions' hysteresis loop area values^a^

P188 (%)	XG (%)	Forward curve		Backward curve		Hysteresis area (Pa s)
		*K* (Pa s^*n*^)	*n*	*K* (Pa s^*n*^)	*n*	
3	0	0.0049 ± 0.0000	1.0400 ± 0.0012	n/a	n/a	n/a
	0.025	0.0167 ± 0.0001	0.9045 ± 0.0017	n/a	n/a	n/a
	0.050	0.0452 ± 0.0007	0.7954 ± 0.0023	n/a	n/a	n/a
	0.075	0.0847 ± 0.0008	0.7065 ± 0.0017	n/a	n/a	n/a
	0.10	0.1482 ± 0.0005	0.6271 ± 0.0011	n/a	n/a	n/a
	0.15	0.3560 ± 0.0033	0.5223 ± 0.0019	n/a	n/a	n/a
	0.20	0.6098 ± 0.0045	0.4537 ± 0.0016	0.5805 ± 0.0054	0.4634 ± 0.0018	4.93 ± 0.88
	0.30	1.4720 ± 0.0071	0.3605 ± 0.0021	1.3115 ± 0.0163	0.3766 ± 0.0033	44.66 ± 7.91
	0.40	2.4005 ± 0.0163	0.3176 ± 0.0013	2.1125 ± 0.0106	0.3380 ± 0.0010	52.74 ± 0.02

a n/a indicates emulsions with no measurable thixotropy (hysteresis loop area); upward and backward flow curves overlapped and no separate descending-phase *K* and *n* values are reported.

Time-dependent rheological behavior was quantified by the hysteresis loop area, obtained by subtracting the area under the backward (descending) flow curve from that under the forward (ascending) curve. The emulsion containing only 3% P188 and no XG exhibited no measurable hysteresis area, confirming the absence of thixotropy, which is consistent with previous studies that emphasize its thermoreversible gelation rather than time-dependent breakdown.^67,68^

Measurable hysteresis loop first appeared at XG concentration of 0.20% and increased further at 0.30% and 0.40%, confirming the onset of time-dependent structural breakdown at higher polysaccharide concentrations (Table 2). These results indicate that structural breakdown and slow recovery become significant once the XG network reaches sufficient entanglement or develops a more integrated internal structure. It is well-known that at sufficiently high concentrations, XG forms a semi-rigid, entangled network with increased zero-shear viscosity and enhanced thixotropic response.^68^ Network formation appears to begin around 0.15–0.20% XG, which corresponds to the emergence of LVR and the first detectable hysteresis loop in this study. Microstructurally, the emulsions appear to contain droplets embedded within an XG-structured continuous phase, with possibly weak depletion flocculation. This loose, shear-sensitive network breaks down when sheared and recovers slowly, producing the observed loop area. The progressive increase in loop area from 0.20% to 0.40% XG reflects the strengthening of this XG-mediated microstructure.^69^

The viscoelastic properties of the P188–XG emulsions were assessed by oscillatory rheological tests. Amplitude sweep tests revealed that only emulsions containing ≥0.15% XG exhibited viscoelastic behavior with a clearly defined LVR, indicating the formation of a weak, internal structure with measurable resistance to deformation. At higher XG concentrations (≥0.20%), the width of the LVR increased, consistent with the formation of a more robust and interconnected microstructure of XG chains and droplets. Yield stress (*τ*_0_) increased progressively with XG concentration, reaching values of 0.371, 0.415, 1.076, and 1.972 Pa for emulsions containing 0.15%, 0.20%, 0.30%, and 0.40% XG, respectively, further supporting the gradual formation of a semi-structured viscoelastic matrix.

**Fig. 9:**
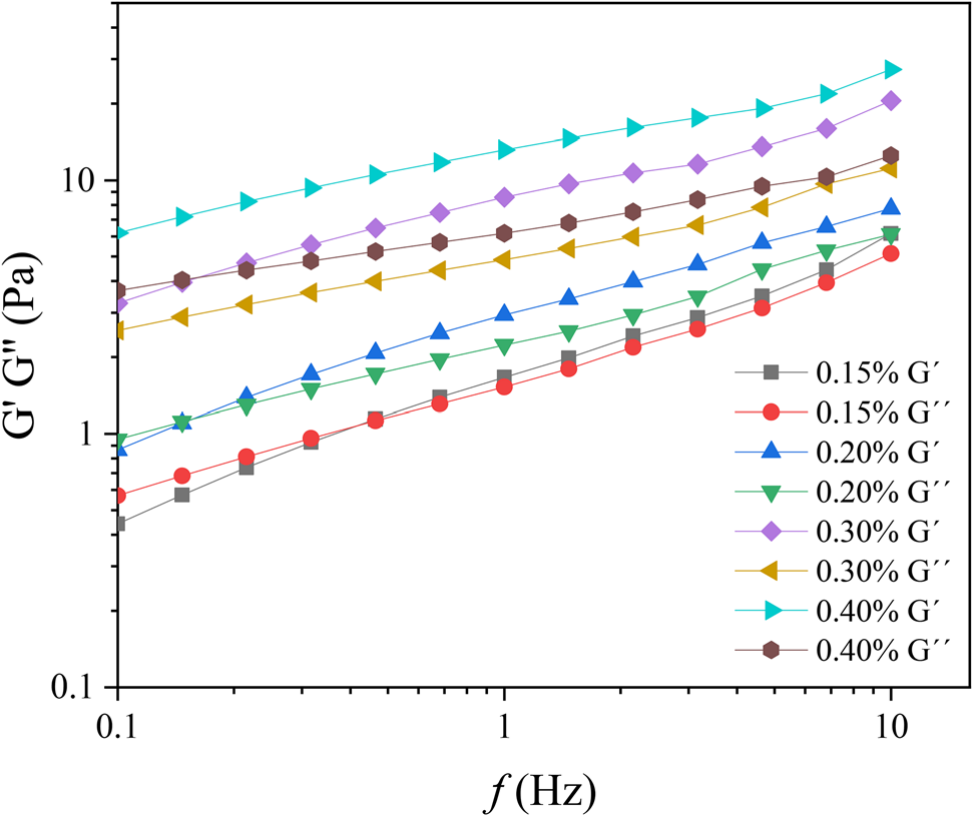
Storage modulus (*G*′) and loss modulus (*G*″) of emulsions with varying xanthan gum (XG) concentrations as a function of frequency (0.1–10 Hz) for formulations containing P188 at 3%.

Frequency sweep tests provided further insight into the viscoelastic nature of the emulsions. For emulsions containing 0.15% and 0.20% XG, *G*″ exceeded *G*′ at low frequencies, but a crossover (*G*′ = *G*″) occurred at intermediate frequencies, beyond which *G*′ predominated (Fig. 9). The crossover point in frequency sweep tests represents the transition between viscous (liquid-like) and elastic (solid-like) responses and is widely used to assess gelation behavior and structural integrity in polysaccharide systems.^70,71^ The crossover point is thus an indicator of weak gel-like behavior, with the system dissipating long-timescale deformations while responding elastically at higher frequencies. At higher XG concentrations (0.30–0.40%), *G*′ was consistently greater than *G*″ throughout the entire frequency range (0.1–10 Hz), demonstrating a predominantly elastic response and confirming the formation of a semi-solid network. Both moduli increased with frequency, in accordance with the behavior of polymer-thickened emulsions.

Overall, the observed progression from viscous-dominated behavior at low XG levels, to weak gel-like behavior at 0.15–0.20% XG, and eventually to predominantly elastic systems at 0.30–0.40% XG corresponds well with the progressive structuring of the continuous phase as XG approaches and surpasses its effective entanglement or critical viscosity concentration. Although the overlap concentration of XG is less than 0.10%, the rheological data show that a deformation-resistant network begins to form above 0.15% XG, with a fully established cohesive matrix composed of polymer chains and dispersed droplets at concentrations of 0.30% and above. This gradual transition is consistent with the stabilization behavior observed in the creaming stability tests, where effective suppression of gravitational separation required XG concentrations of 0.20% and higher.

Rheological data indicate that the combination of P188 and XG yields emulsions that do not form a rigid gel but rather a weak, semi-structured viscoelastic matrix capable of resisting flow and deformation. This rheological behavior directly supports the improved creaming stability observed at higher XG concentrations, as elastic-dominated emulsions exhibit better gravitational stability. P188 enhances this effect by providing sterically stabilized droplets and a more uniform droplet size distribution, thereby facilitating the formation of a robust but deformable network structure.^72,73^

In practical terms, these results are highly relevant for pharmaceutical and cosmetic formulations. Emulsions containing P188 and natural gums exhibit excellent spreadability, improved bioadhesion, and the potential for controlled release.^74,75^ The rheological tunability achieved by modulating XG concentration allows tailoring of flow and viscoelastic behavior of the emulsions to meet the demands of particular applications.

## 4 Conclusions

The surface and interfacial tension measurements indicated that there are no attractive interactions between P188 and XG in the investigated systems. The visual observations of emulsions during 21 days of storage showed that the concentration of both P188 and XG influenced the creaming stability of the emulsions. While the higher concentration of P188 increased the stability, the rise in XG concentration had a more complex influence on creaming. With the increase in the polysaccharide concentration, the gravitational stability initially decreased, most likely due to depletion flocculation that arose with the addition of the non-adsorbing macromolecule. The most stable emulsions were formed at XG concentrations of 0.20% and higher, indicating that the critical viscosity concentration was reached or surpassed. These concentrations of the polysaccharide also prevented significant change in the Sauter mean droplet size and size distribution in the emulsions during 21 days of storage. The emulsions with a lower concentration of XG, *i.e.*, below 0.10%, showed the most observable change in the Sauter mean diameter of droplets and size distribution, most probably due to the effect of depletion flocculation and lower viscosity of the continuous phase. The rheological characterization indicated that 3% P188 and XG at concentrations above 0.20% produce emulsions with a semi-structured viscoelastic matrix composed of a network of polymer chains and dispersed droplets, capable of resisting flow, deformation, and gravitational instability.

## Author contributions

Dejan Ćirin: conceptualization, writing – original draft, visualization, investigation, formal analysis. Nebojša Pavlović: writing – original draft, formal analysis, investigation, data curation. Ivana Nikolić: visualization, validation, methodology. Jovana Milutinov: visualization, validation, software. Dragana Zaklan: data curation, software. Milica Atanacković Krstonošić: writing – review & editing, methodology. Veljko Krstonošić: writing – review & editing, supervision, resources.

## Conflicts of interest

The authors declare that they have no known competing financial interests or personal relationships that could have appeared to influence the work reported in this paper.

## Data Availability

The data used to support the findings of this study are included in the article.
